# Water-soluble variant of human Lynx1 induces cell cycle arrest and apoptosis in lung cancer cells via modulation of α7 nicotinic acetylcholine receptors

**DOI:** 10.1371/journal.pone.0217339

**Published:** 2019-05-31

**Authors:** Maxim Bychkov, Zakhar Shenkarev, Mikhail Shulepko, Olga Shlepova, Mikhail Kirpichnikov, Ekaterina Lyukmanova

**Affiliations:** 1 Shemyakin-Ovchinnikov Institute of Bioorganic Chemistry RAS, Moscow, Russian Federation; 2 Moscow Institute of Physics and Technology, Dolgoprudny, Moscow Region, Russian Federation; 3 Faculty of Biology, Lomonosov Moscow State University, Moscow, Russian Federation; University of Pécs Medical School, HUNGARY

## Abstract

Lynx1 is the first three-finger prototoxin found in the mammalian central nervous system. It is a GPI-anchored protein modulating nicotinic acetylcholine receptors (nAChRs) in the brain. Besides the brain, the Lynx1 protein was found in the lung and kidney. Endogenous Lynx1 controls the nicotine-induced up-regulation of the expression of α7 type nAChRs in lung adenocarcinoma A549 cells as well as the cell growth. Here, we analyzed the Lynx1 expression in the set of human epithelial cells. The Lynx1 expression both at the mRNA and protein level was detected in normal oral keratinocytes, and lung, colon, epidermal, and breast cancer cells, but not in embryonic kidney cells. Co-localization of Lynx1 with α7-nAChRs was revealed in a cell membrane for lung adenocarcinoma A549 and colon carcinoma HT-29 cells, but not for breast adenocarcinoma MCF-7 and epidermoid carcinoma A431 cells. The recombinant water-soluble variant of Lynx1 without a GPI-anchor (ws-Lynx1) inhibited the growth of A549 cells causing cell cycle arrest via modulation of α7-nAChRs and activation of different intracellular signaling cascades, including PKC/IP_3_, MAP/ERK, p38, and JNK pathways. A549 cells treatment with ws-Lynx1 resulted in phosphorylation of the proapoptotic tumor suppressor protein p53 and different kinases participated in the regulation of gene transcription, cell growth, adhesion, and differentiation. Externalization of phosphatidylserine, an early apoptosis marker, observed by flow cytometry, confirmed the induction of apoptosis in A549 cells upon the ws-Lynx1 treatment. Our data revealed the ability of ws-Lynx1 to regulate homeostasis of epithelial cancer cells.

## Introduction

Nicotinic acetylcholine receptors (nAChRs) are ligand-gated ion channels responsible for signal transduction in the central and peripheral nervous systems and in the neuromuscular junctions [1]. nAChRs are homo- or hetero-pentamers, composed of α and non-α subunits. nAChRs were also found in non-excitable cells, like immune and epithelial cells [2]. These non-neuronal receptors participate in proliferation, differentiation, migration and apoptosis of epithelial cells, in control of inflammation, as well as in regulation of gene transcription [3–5]. The expression of different nAChR subunits was described in epithelial human cancers, e.g. in lung cancer, mesothelioma and colon carcinoma [6].

Higher order animals produce endogenous proteins from the Ly6/uPAR family, which share structural homology with snake α-neurotoxins and modulate function of nAChRs [7,8]. Some of them (e.g. Lynx1) are membrane-tethered by a GPI-anchor near their receptors in the brain [9]. Other Ly6/uPAR proteins (e.g. SLURP-1 and SLURP-2) are secreted by immune cells [10], sensory neurons [11], and epithelial cells [12] and participate in the development of a number of skin diseases including Mal de Meleda [13,14], psoriasis [15] and cancer [16,17]. Recently we showed that SLURPs affect the expression of α7 type nAСhRs (α7-nAChRs) in epithelial cancer cells [18] and inhibit their growth [18,19].

Lynx1 is a GPI-tethered protein co-localized with α7- and α4β2-nAChRs in the brain [9], although a soluble form of Lynx1 is also detected in the cerebrospinal fluid [20]. Lynx1 is important for activity of α6 containing nAChRs [21], modulates the expression of α3β4 and α3β4α5 nAChRs on a plasma membrane and reduces the magnitude of ACh-evoked macroscopic currents through α3β4 receptors [22]. Also, Lynx1 mediates nociception in dorsal raphe nucleus by modulation of the low sensitivity α4β2 nAChRs [23]. Except the nervous system, the Lynx1 protein expression was also found in the lung and kidney, but in much lower amounts [9,20,24]. The decreased Lynx1 level was revealed in lung cancer cells in comparison with normal cells [25]. Silencing of the *LYNX1* gene in lung carcinoma A549 cells leads to the significant increase in the cancer cell growth [26]. *LYNX1* expression was also found in normal human epidermal keratinocytes [27]. Altogether, these data indicate that Lynx1 could be expressed not only in the nervous system, but also could participate in the regulation of epithelial cell homeostasis.

Here, we studied the Lynx1 expression in human cells of epithelial origin (normal oral keratinocytes Het-1A, embryonic kidney cells HEK-293T, epidermoid carcinoma A431, breast adenocarcinoma MCF-7, lung adenocarcinoma A549 and colon carcinoma HT-29). Additionally, we investigated the influence of the recombinant soluble variant of human Lynx1 without the GPI-anchor (ws-Lynx1, [28]) on growth and apoptosis of A549 cells. Data obtained revealed the ability of ws-Lynx1 to regulate homeostasis of cancer cells via interaction with α7-nAChRs, causing the cell cycle arrest and activation of proapoptotic intracellular cascades.

## Materials and methods

### Materials

Recombinant ws-Lynx1 and its R38A mutant were produced in *E*. *coli* as described previously [29]. Recombinant analogue of neurotoxin II from *Naja oxiana* was obtained as in [30]. The purity and homogeneity of the protein preparations were confirmed by HPLC, MALDI-MS, and SDS-PAGE. Disulfide bond formation was confirmed in the reaction with Ellman’s reagent (Sigma-Aldrich, USA). The correct spatial structure was confirmed by NMR spectroscopy.

Bicuculline, gefitinib, AG-825, timolol, methyllicaconitine (MLA), PD98059, SP600125, SB203580, Bay-11-7082 and Go 6983 were the products of Tocris. Мecamylamine hydrochloride (Mec), nicotine, wortmannin, and propidium iodide were obtained from Sigma-Aldrich. JSH-23 was product of Santa-Cruz (USA). S31-201, 285986-31-4 and xestospongin B were from Calbiochem (USA).

Six adult male Wistar rats (230–283 g) were obtained from the Puschino Breeding Center (Moscow region, Russia) and housed 2 animals per cage. Animals were kept on a 12:12-hour light:dark cycle provided with a standard rodent diet and water *ad libitum*. The animals were acclimatized for at least 7 days after arrival before experiments began. They were anesthetized with isoflurane and then decapitated. The brain was dissected and stored at -70°C. For the primary culture of cortical neurons newborn Wistar rats were used. The study was performed in accordance with the guidelines set forth by the European Communities Council Directive of November 24, 1986 (86/609/EEC). The study was approved by the institutional commission of the Shemyakin-Ovchinnikov Institute of Bioorganic Chemistry RAS for the control of the maintenance and use of animals (protocol #222 from 13 February 2018).

### Cell cultivation

Human epidermoid carcinoma cells A431 (ATCC, USA), human breast adenocarcinoma cells MCF-7 (ATCC, USA), human lung adenocarcinoma A549 cells (ATCC, USA), and HEK-293T cells (Institute of Cytology RAS, Russia) were grown (37°C, 5% CO_2_) in a DME medium with phenol red (PanEco, Russia), 10% fetal calf serum (Thermo Fisher Scientific, USA) and 2 mM L-glutamine (PanEco), abbreviated below as the complete medium. Human colon carcinoma HT-29 cells (ATCC) were grown in a RPMI-1640 medium, supplemented with 10% fetal calf serum and 2 mM L-glutamine. Human immortalized oral Het-1A keratinocytes (ATCC) were cultivated in a BEB medium (Lonza, Switzerland). Before cell subculturing and performing experiments the culture flasks and plates were pre-coated with a mixture of 0.01 g/L fibronectin (Sigma-Aldrich), 0.03 g/L bovine collagen type I (Sigma-Aldrich) and 0.01 g/L bovine serum albumin (Sigma-Aldrich) dissolved in the culture medium. All types of the cells were subcultured twice per week. The primary culture of cortical neurons were obtained as previously described [31]. Neurons were cultured in a Neurobasal-A medium (Gibco, USA) supplemented with Glutamax (Life Technologies, USA) and B27 (Gibco) (so called complete medium) on polylysine-coated cover glasses in 24-well plates for two days, then Cytarabin (20 μM) (Sigma-Aldrich) was added to inhibit growth of glial cells and the culture was cultivated for 12 days with media change every 4 days and then taken to experiments.

### Real-time PCR

To extract total mRNA from the whole rat brain extract, 1 ml of ExtractRNA reagent (Evrogen, Russia) was added to the frozen rat brain, and then the brain was rapidly homogenized. Total mRNA from cultured cells was also extracted using ExtractRNA reagent according to manufacturer’s instructions. Then mRNA from the rat brain or cultured cells was treated by DNAse I (Sigma-Aldrich) and purified using CleanRNA Standard kit (Evrogen). Total cDNA was synthesized using MMLV RT kit (Evrogen) in accordance to the manufacturer's protocol. After that real-time PCR was performed with the primers described in the S1 Table, and ready-to-use qPCR mix with SYBR Green I and reference ROX fluorescent dyes (Evrogen). Briefly, 12.5 μL of the diluted cDNA sample produced from 1 μg of total RNA was added to 7.5 μL of the PCR master mix and 5 μL of 5× qPCR mix-HS SYBRHigh ROX (Evrogen). Negative controls contained all the components of the PCR mixture except cDNA and gave no signal. All PCR reactions were performed using DT96 Prime real-time detection thermal cycler (DNA Technology, Russia). Data was analyzed by theΔCt method [32]. Expression level of the nAChR subunits was normalized to the expression level of the β-actin house-keeping gene.

### Confocal fluorescent microscopy

Het-1A, A431, MCF-7, A549, HT-29, and HEK-293T cells were seeded on 8-chamber slide-flask one day before experiment in the complete medium. Then a silicone chamber was removed from a glass slide, and the slide with attached cells was incubated for 2 h in CO_2_ incubator with antibodies against Lynx1 (rabbit anti-Lynx1 Abcam, USA, ab125035, 1:1000, 0.76 μg/ml) and with antibodies against α7-nAChRs (mouse anti-α7-nAChR; Sigma, USA, M220, 1:2500, 2 μg/ml). After that, the cells were washed 3 times by the Earle's balanced salt solution (EBSS) and fixed with 1% formaldehyde solution (20 min, 4°C). Then the incubation with the secondary goat anti-rabbit AlexaFluor 488 labeled IgG (Jackson Immunoresearch, USA, 711-545-152, 1:500, 2 μg/ml) for visualization of Lynx1 and anti-mouse Alexa-594-labelled IgG (Jackson Immunoresearch, 715-585-150, 1:500, 2 μg/ml) for visualization of α7-nAChRs was carried out during 1 h. Neurons were cultivated on cover glasses and stained as other cells. The cell nuclei were visualized by Hoechst 33342 (0.1 μg/ml, PanEco). After washing 3 times by EBSS, the cells were embedded in glycerol. The cover glass was immobilized by nail polish and was observed under ×60 oil-immersion objective of Nikon C1+ (TE2000E) inverted confocal laser scanning microscope (Nikon, Japan). The laser power and detector voltages were equal for all the experimental sessions.

The cells stained only by the secondary antibodies are presented in S1 and S2 Figs. Specificity of anti-Lynx1 antibodies was analyzed using ws-Lynx1. Briefly, 6.7 nM of antibodies were incubated with 1 μM ws-Lynx1 for 1 h, and then A549 cells were stained with this mixture and the primary mouse anti-α7-nAChR antibodies. Then, the cells were stained by the secondary anti-rabbit AlexaFluor 488 labeled IgG and anti-mouse Alexa-594-labelled IgG as described above (S3 Fig).

### Growth of A549 cells

To study the effect of ws-Lynx1 on the A549 cell growth, the cells were seeded in 96-well cell culture plates in the complete medium (0.5×10^4^ cells/well) and grown for 24 h. Thereafter ws-Lynx1 from the 100% DMSO stock solution was diluted in the complete medium and added to the cells at different concentrations for further incubation during 24, 48 or 72 h. Every 24 h the cell medium was aspirated and replaced by the fresh complete medium, containing pre-dissolved ws-Lynx1 in the same concentration as in the first day. The final DMSO concentration did not exceed 0.1%. The added DMSO did not influence the cell growth as was checked in additional experiments.

For co-application of ws-Lynx1 with different compounds, A549 cells were pre-incubated with 2 μM bicuculline, 10 nM gefitinib, 150 nM AG 825, 2 nM timolol, 1 μM Mec, 1 μM MLA, 10 nM nicotine, 1 μM PD98059, 100 nM SP600125, 1 μM SB203580, 3 nM wortmannin, 10 μM Bay11-7082, 1 μM JSH-23, 100 μM S31-201, 10 μM 285986-31-4, 1 μM Go 6983 or 10 μM xestospongin B for 30 min. Then the cells were rinsed twice with the fresh complete medium, 1 μM ws-Lynx1 and the respective compound were added to the complete medium, and the cells were incubated further for 72 h. Every 24 h the cell medium was aspirated and replaced by the fresh complete medium, containing pre-dissolved ws-Lynx1 and the respective compound at the same concentration as in the first day.

Cell growth was assayed using the WST-1 colorimetric test as described elsewhere [33]. WST-1 (water soluble tetrazolium salt 1, Santa Cruz) and 1-m-PMS (1-methoxy-5-methylphenazinium methyl sulfate, Santa Cruz) were added to the cells in concentrations of 0.25 mM and 5 μM, respectively, for 2 h, and the formation of colored product was measured in the 450 nm spectral range using microplate reader Bio-Rad 680 (Bio-Rad, USA).

### Cell cycle arrest in A549 cells

Cells we seeded in 6-well culture plates (12.5×10^3^ cells per well) and incubated with 1 μM ws-Lynx1 for 24, 48, or 72 h as described above. Then the cells were detached from the wells by trypsine, washed with EBSS, and fixed in ice-cold 70% ethanol for 4 h. After fixation, the cells were washed twice by EBSS, and DNA was extracted by 5 min incubation with the DNA extraction buffer (200 mM Na_2_HPO_4_ with 0.004% Triton X-100, pH 7.8). Then the cells were washed by EBSS, resuspended in the DNA staining solution (EBSS, 50 mg/ml propidium iodide, 0.2 mg/ml DNase free RNAse), and analyzed by FACSCalibur flow cytometer (Becton Dickinson, USA). The data were analyzed using Flowing 2.5.1 software (Turku Centre for Biotechnology, Finland).

### Silencing of α7-nAChR

To block the expression of native α7 receptors, A549 cells were transfected with siRNA to α7-nAChRs (siRNA duplex was formed by GGAAGCUUUACAAGGAGCUGGUCAA and UUGACCAGCUCCUUGUAAAGCUUCC synthetic oligonucleotides (Synthol, Russia), [34]). Cells were seeded in a 6-well culture plates (1×10^5^ cells per well) and grown for 24 h. Then siRNA (1 μg per well) was diluted in 100 μl transfection buffer (Pan-Biotech, Germany), incubated for 5 min and mixed with 15 μl pre-diluted PanFect A-plus transfection reagent (Pan-Biotech, Germany). The final mixture was incubated for 30 min and added to the A549 cells. The cells were incubated in CO_2_-incubator during 4 h and the cell media was replaced by the fresh one. After the 48 h incubation, the cells were detached by Versene solution and divided onto the two parts. The first part was incubated with TRITC-labelled α-bungarotoxin (Sigma, USA), and the expression of functional α7-nAChRs on the cell membrane was analyzed by flow cytometry. The second part of the cells was seeded on 96-well culture plates (5×10^4^ cells per well) and incubated with 1 μM ws-Lynx1 for 72 h as described above. Cell viability was analyzed by the WST-1 assay.

### Kinase phosphorylation upon ws-Lynx1 application

To investigate the Lynx1 influence on the cellular signal transduction in A549 cells, we used a Proteome Profiler antibody array (ARY003B, R&D Systems, USA), which allowed a simultaneous detection of phosphorylation of different kinases. The cells were seeded in T25 cell culture flasks and incubated with 1 μM ws-Lynx1 (from the 100% DMSO stock) or an equal DMSO amount in control flasks. The cells were grown for 72 h as described above. After the 72 h incubation the cells were detached by scrapping and lysed by 400 μl of Lysis buffer, provided with the Proteome Profiler antibody array. After the lysate centrifugation, the supernatants were collected, and the human phospho-kinase array was performed according to the manufacturer protocol. Briefly, the array membranes with immobilized capture antibodies were blocked, incubated with the cell lysates overnight and then consequently incubated with biotinylated detection antibodies and streptavidin-HRP. The HRP signal was detected by ECL substrate using Bio-Rad Versa-Doc 4000 imager (Bio-Rad). The spots intensity was quantified by Image J software (NIH).

### Study of the A549 cells apoptosis by flow cytometry

To investigate apoptosis in A549 cells, we used Annexin V for detection of the phosphatidylserine externalization, one of the early apoptosis markers. Briefly, A549 cells were seeded on a 35-mm Petri dish (1×10^5^ cells/dish) and incubated with 1 μM ws-Lynx1 for 72 h as described above. After incubation, the cells were detached by the Versene solution and washed in PBS. Then, the cells were incubated with Annexin V conjugated to phycoerythrin (PE) (A35111, Thermo Fisher Scientific) and washed by PBS. Finally, the cells were analyzed on BD FACSCalibur flow cytometer (Becton Dickinson). The data were analyzed using Flowing 2.5.1 software.

## Results and discussion

### Expression of Lynx1 and nAChR genes in human non-neuronal cells

**Fig 1:**
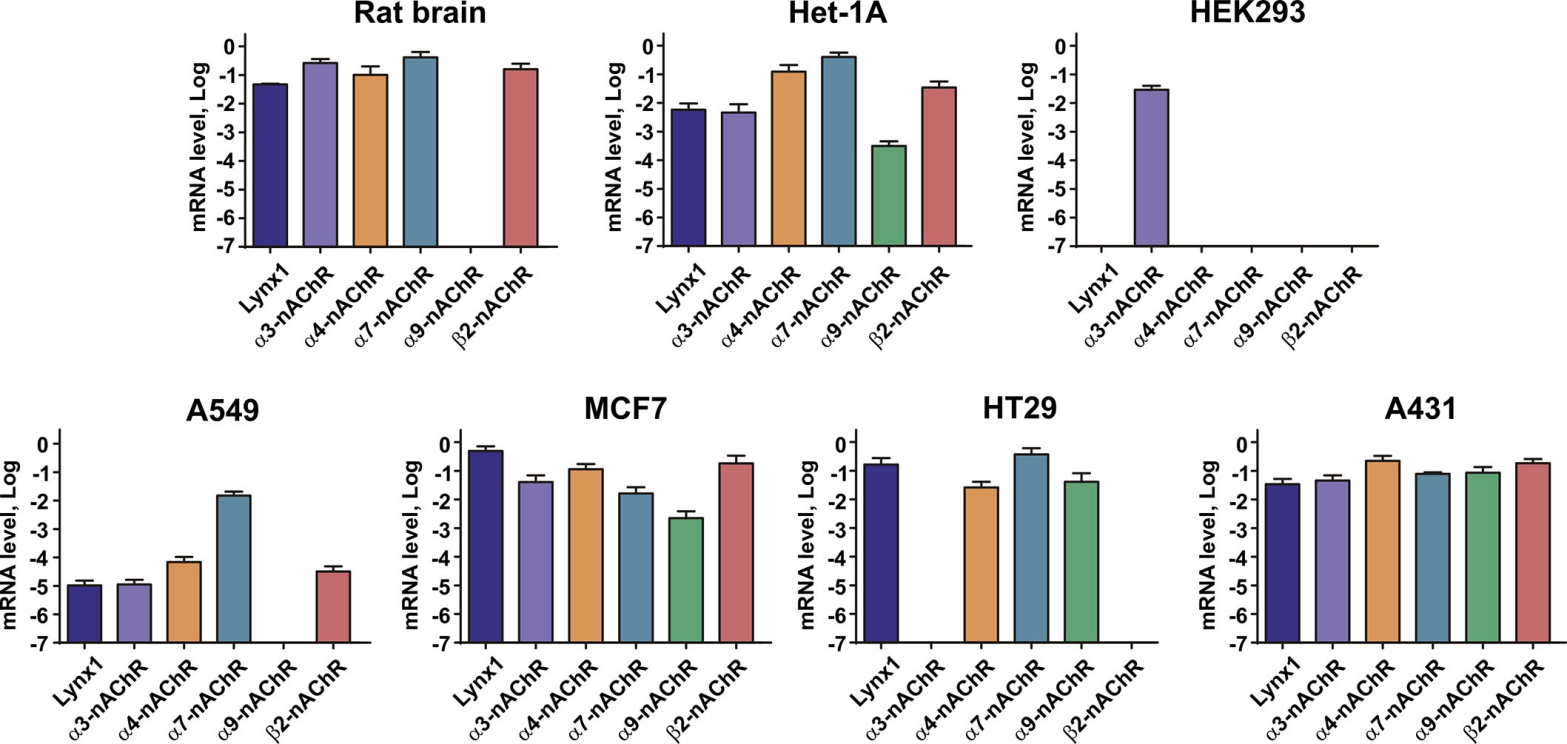
Expression profile of α3, α4, α7, α9, and β2 nAChR subunits genes in epithelial cells. 5 different portions of cells in triplicates were analyzed (n = 15). Measured data were normalized to the expression level of housekeeping gene β-actin by the ΔCt method and presented as mean ± SEM on a logarithm scale. The β-actin gene expression level at this scale corresponds to zero.

In our work, we used several lines of epithelial cells. The rat brain extract was used as a positive control. Using real-time PCR, we found the different expression profile of the *α3*, *α4*, *α7*, *α9*, and *β2* nAChR subunits genes in the studied cells (Fig 1).

In general, our data agree well with the previous reports, although some new details were revealed. Similarly to the known localization of different nAChRs subunits in the mammal brain [35], we observed the expression of all studied subunits genes (except *α9*) in the rat brain extract. The gene expression of the *α7*, *α9*, and *β4* nAChR subunits [36] and the protein expression of α9 nAChR subunit [37] in breast adenocarcinoma MCF-7 cells were previously reported. The Human Protein Atlas (HPA) database also describes expression of the *α3* gene in this cell line, while our study also detected the expression of α4 and β2 genes. Previously, the analysis of protein expression in lung adenocarcinoma A549 cells revealed the presence of the α3, α4, α7, α9, β2, and β4 nAChR subunits [38]. Contrary to that, we did not observe the expression of the *α9* nAChR subunit. Colorectal carcinoma HT-29 cells were demonstrated the expression of the *α4*, *α5*, *α7*, and *β1* genes [39], while we also found the expression of the *α9* nAChR subunit. It was previously shown that epidermoid carcinoma A431 cells express the *α7* nAChR gene [40], and the expression of the *α3* gene was reported in the HPA database, while we additionally detected the expression of the *α4*, *α9*, and *β2* genes. We found the gene expression of the *α3*, *α4*, *α7*, *α9* and *β2* nAChR subunits in Het-1A cells that corresponds to the previous study [3]. Similarly to the data from the HPA database, in embryonic kidney HEK-293T cells we observed the expression only of the *α3* subunit. It should be mentioned, that the expression of nAChR subunits at the mRNA level, could not correlate with the expression of the functional receptors composed from these subunits on a cellular membrane. Thus, the observed gene expression of the nAChR subunits does not indicate the real expression of the corresponding receptors.

All the cells studied, except HEK-293T, demonstrated the significant level of the *LYNX1* expression (Fig 1). It contradicts the previous data about the *LYNX1* expression in the kidney [9]. Probably, the LYNX1 expression depends on the stage of the development and rises after birth. In agreement with this hypothesis, no *Lynx1* expression was found in the rat cortex and hippocampus up to the 14th postnatal day [20], and the significant increase of the *Lynx1* mRNA level in the mouse cerebellum was observed from P0 to P20 days [41]. Similarly, *LYNX1* is absent in the fetal breast, skin, and colon tissues, but is expressed in adult tissues (68 FANTOM5 RNA-seq dataset, https://www.ebi.ac.uk/gxa).

According to the HPA database, the *LYNX1* expression was reported in epidermoid carcinoma A431 and lung adenocarcinoma A549 cells, and in HACAT immortalized keratinocytes, however it was not detected in breast adenocarcinoma MCF-7 cells. Probably, these discrepancies in the *LYNX1* expression according to the different sources are due to the different approaches and lab conditions used for mRNA extraction and detection.

### Lynx1 is co-localized with α7-nAChRs in epithelial cells

**Fig 2:**
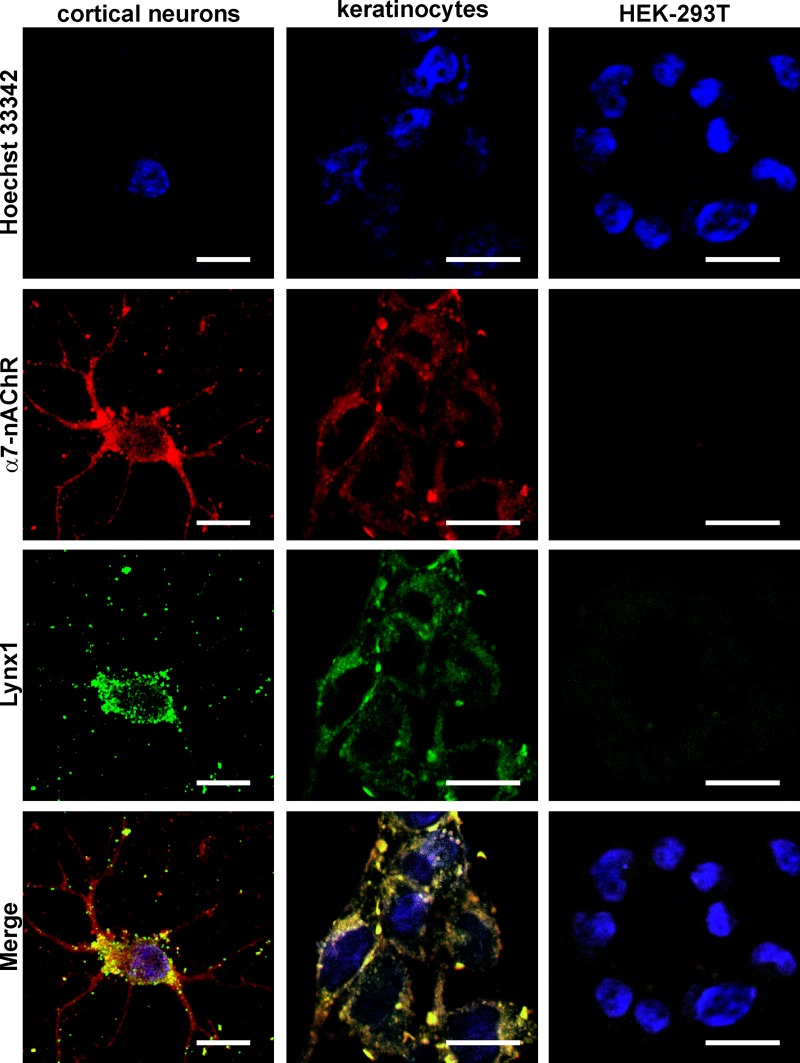
Colocalization of endogenous Lynx1 and α7-nAChRs in primary cortical neurons, oral Het-1A keratinocytes and HEK-293T cells. Cells were sequentially incubated with the rabbit anti-Lynx1 and mouse anti-α7-nAChR primary antibodies and with secondary anti-rabbit Alexa-488 labeled IgG (green) and anti-mouse Alexa-594 labeled antibodies (red). Cell nuclei were visualized by Hoechst 33342 (blue). Scale bar 10 μm.

**Fig 3:**
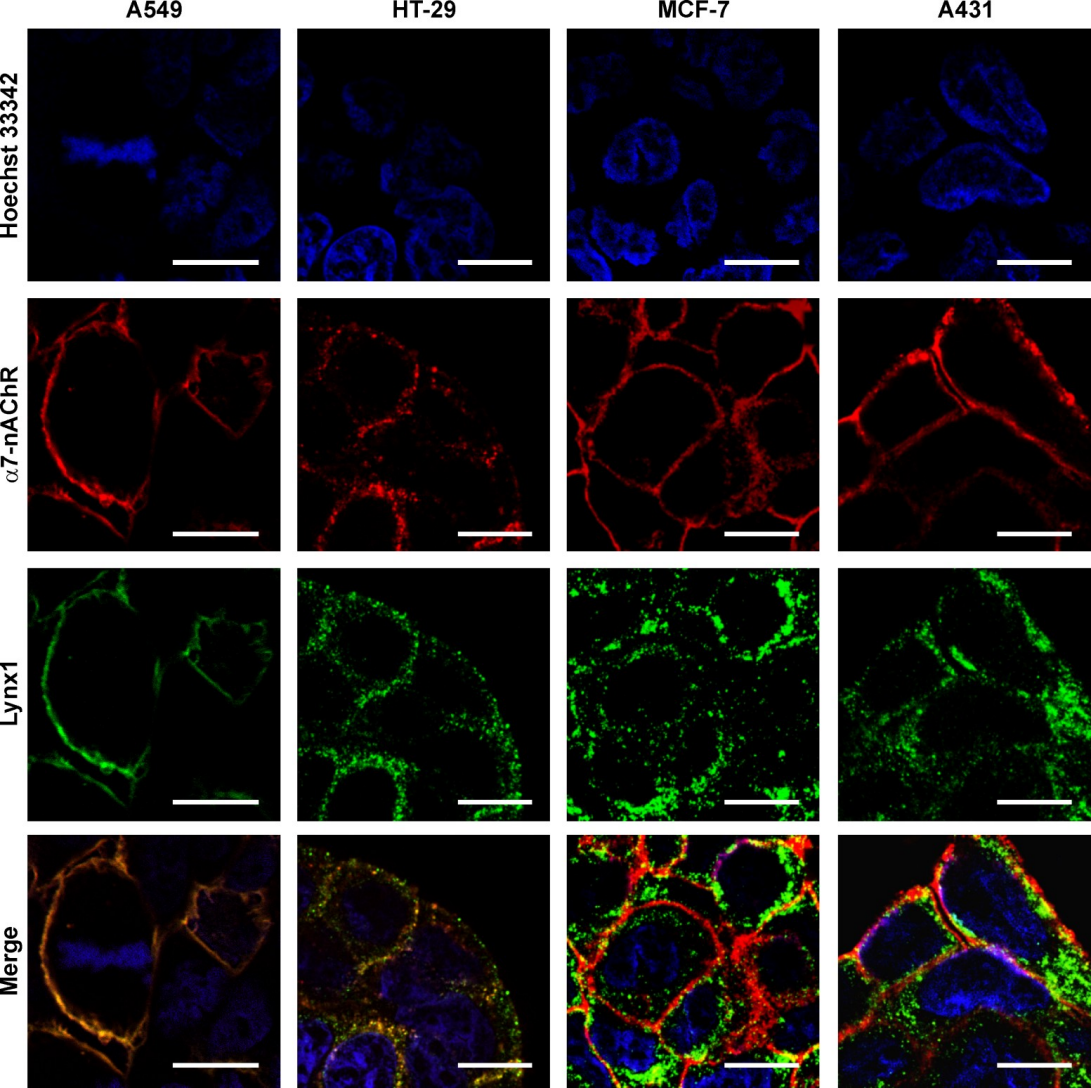
Colocalization of endogenous Lynx1 and α7-nAChRs in A549, HT-29, MCF-7, and A431 cells. Cells were sequentially incubated with the rabbit anti-Lynx1 and mouse anti-α7-nAChR primary antibodies and with secondary anti-rabbit Alexa-488 labeled IgG (green) and anti-mouse Alexa-594 labeled antibodies (red). Cell nuclei were visualized by Hoechst 33342 (blue). Scale bar 10 μm.

To prove the Lynx1 and α7 nAChR subunit expression at a protein level, we used confocal fluorescent microscopy. In agreement with the observed gene expression patterns, no Lynx1 and α7-nAChR at the protein level were detected in HEK-293T cells, while in other studied cells the expression of both proteins was observed (Figs 2 and 3). For the best of our knowledge, this is the first report about the Lynx1 expression in colon and breast cancer cells as well as in Het-1A oral keratinocytes.

To study the possible target of Lynx1, we analyzed its co-localization with α7-nAChRs. Primary culture of cortical neurons was used as a positive control. In neurons, α7-nAChRs were expressed in the soma and dendrites, while Lynx1 was observed mainly in the soma (Fig 3). Evident membrane co-localization of Lynx1 and α7-nAChRs was observed only in A549 and HT-29 cells (Fig 3). Similar co-localization of Lynx1 and α7-nAChRs was previously demonstrated in developing lung tissue [24] as well as in adult lung epithelium [26]. Surprisingly, in MCF-7 and A431 cells Lynx1 and α7-nAChRs demonstrated different expression pattern with accumulation in intracellular punctuates and cell membrane, respectively (Fig 3). Our results point on a widespread Lynx1 expression in cells of different origin and indicate that Lynx1 could act not only as the neuromodulator in the nervous system but also could be implicated in physiological processes in epithelial cells.

### Ws-Lynx1 down-regulates the viability of lung cancer cells and abolishes nicotine-induced cell growth stimulation

α7-nAChR is the main receptor which mediate the proliferative effects of nicotine in cancer cells [6]. Activation by nicotine could increase the α7-nAChR expression and stimulate growth and migration of cancer cells [6,26,42]. Nicotine is known to enhance expression of hypoxia-related molecules such as HIF-1a in A549 cells stimulating secretion of different growth factors and their resistance to chemotherapy [42]. Also, activation of α7-nAChRs in endothelial cells by nicotine leads to tumor vascularization through the release of VEGF and FGF [42]. Another aspect of nicotine action on tumor development is its ability to induce self-renewal of cancer stem cells by a simultaneous increase of stress neurotransmitters production and decrease of GABA production [43]. On the other hand, endogenous Lynx1 suppresses the nicotine-induced up-regulation of the α7-nAChR expression [26,44]. Moreover, the up-regulation of the Lynx1 expression in lung carcinoma A549 cells by lentiviral transduction decreases cancer cell proliferation [26]. Based on these observations, we decided to evaluate the ability of the recombinant water-soluble variant of Lynx1 (ws-Lynx1) to control the growth of A549 cells.

**Fig 4:**
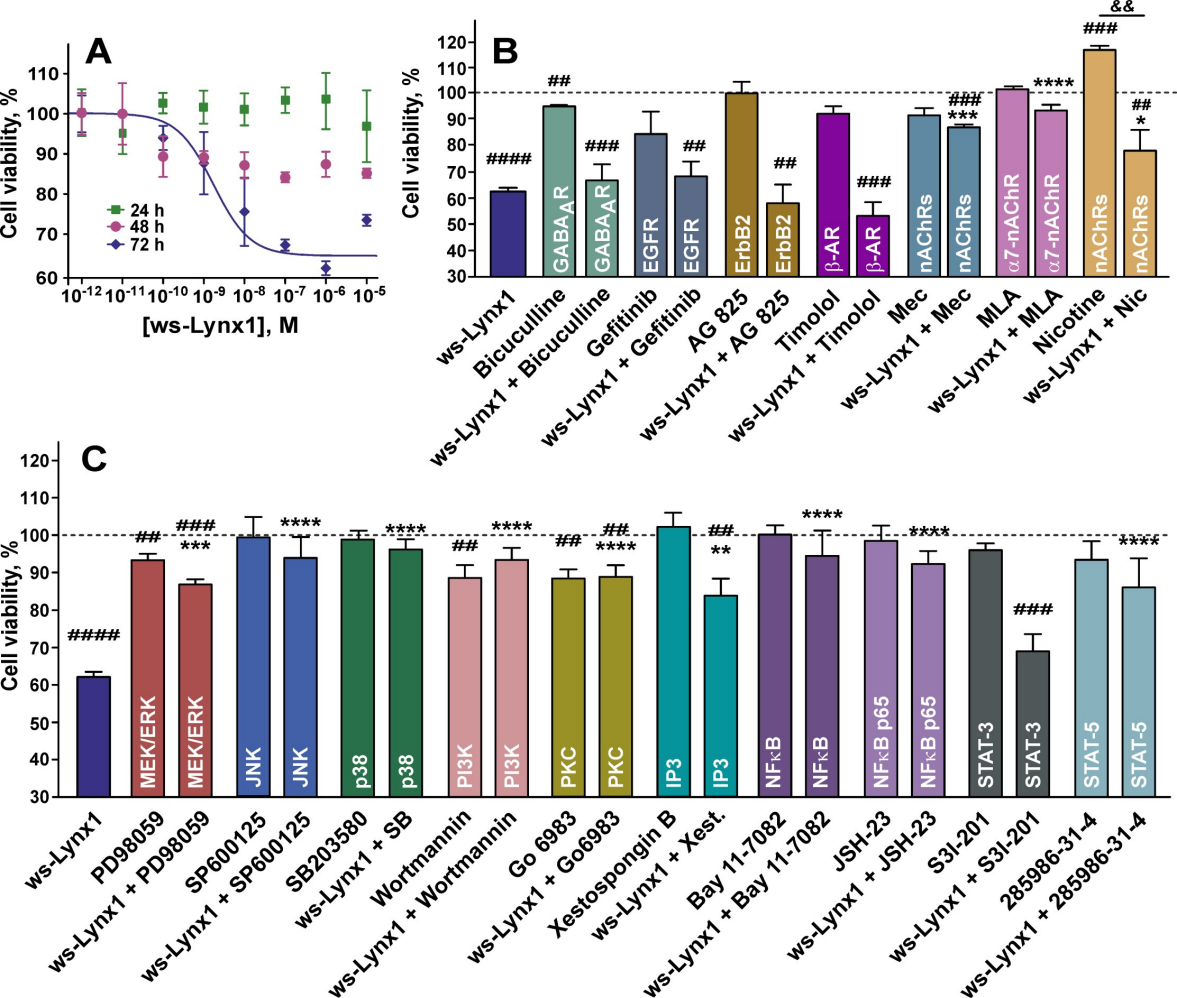
Influence of ws-Lynx1 on the growth of A549 cells. (A). Effects of ws-Lynx1 on the growth of A549 cells during 24-, 48- and 72-hour incubation. The data are expressed as % of the control (untreated cells). Each point is a mean ± S.E.M. of 4–10 independent experiments. (B). The influence of ws-Lynx1, the inhibitors of different receptors, and nicotine on the viability of A549 cells. (C). Effects of ws-Lynx1 and inhibitors of intracellular signaling cascades on the A549 cells viability. Cells in (B,C) were incubated with ws-Lynx1 and other compounds during 72 hours as described in the Materials and Methods section. The data are mean ± SEM, n = 4–8; ^*##*^ (p<0.01), ^*###*^ (p<0.001), and ^*####*^ (p<0.0001) indicate the significant difference from the control (100%) by a two-tailed one sample t-test; ^*&&*^ (p<0.01) indicates the significant difference between the “Nicotine” and “ws-Lynx1 + Nicotine” groups by a two-tailed t-test; * (p<0.05), ** (p<0.01), *** (p<0.001), and **** (p<0.0001) indicate the significant difference between the “ws-Lynx1” and “ws-Lynx1 + agonist/inhibitor” groups by One-way ANOVA followed by a Dunnet’s *post hoc* test. The control level (100% of viable cells) corresponds to the untreated cells.

Prolonged 72 h incubation of A549 cells with ws-Lynx1 resulted in the pronounced concentration-dependent inhibition of the cell growth with EC_50_ of 1.9 ± 0.3 nM (Fig 4A). The number of viable cells was reduced up to 66.7 ± 1.3% relative to the control. Shorter 48 h administration resulted in the weak but significant reduction of the number of viable cells, while no significant effect was observed after 24 h incubation (Fig 4A). It should be mentioned, that similar antiproliferative activity on A549 cells for the recombinant analogue of the other human Ly6/uPAR protein,—SLURP-1, also acting on α7-nAChRs was recently reported [18]. However, recombinant SLURP-1 demonstrated the significant antiproliferative effect already after 24 h incubation [18]. This revealed differences in the mechanisms of the Lynx1 and SLURP-1 action. Probably, the faster SLURP-1 effect is connected with the secretion of endogenous SLURP-1 from the intracellular depot in response to the recombinant SLURP-1 application. This results in the significant increase of the SLURP-1 concentration in the cell medium after several hours [18]. Contrary, endogenous Lynx1 is a membrane-tethered protein, and its concentration in the cell medium cannot be enhanced by secretion. The delayed effect of ws-Lynx1 on the cell viability implies the presence of a multi-step response, which could involve (1) changes in the expression of auto- paracrine regulators like SLURPs, growth factors, cytokines (like TNFα) etc; and (2) activation of the respective receptors and intracellular signaling cascades, which control cell growth and proliferation. Moreover, as it will be shown below, ws-Lynx1 simulates proapoptotic signaling cascades, while no signs of apoptosis were previously found in the SLURP-1 study [18].

We also tested whether ws-Lynx1 could affect the nicotine-induced A549 cell growth (Fig 4B). Incubation of A549 cells during 72 hours with 10 nM nicotine significantly increased the A549 cell proliferation (~118% of viable cells relative to the control). Contrary, co-application of nicotine with 1 μM ws-Lynx1 canceled this effect, and reduced the number of viable cells up to ~79% relative to the control (Fig 4B). The data obtained are in line with the previous report about the action of membrane-tethered Lynx1 in A549 cells [26]. Thus, we can conclude that the water-soluble variant of Lynx1 suppresses the nicotine-induced cancer cell growth *in vitro*.

### Ws-Lynx1 induces cell cycle arrest in A549 cells

**Fig 5:**
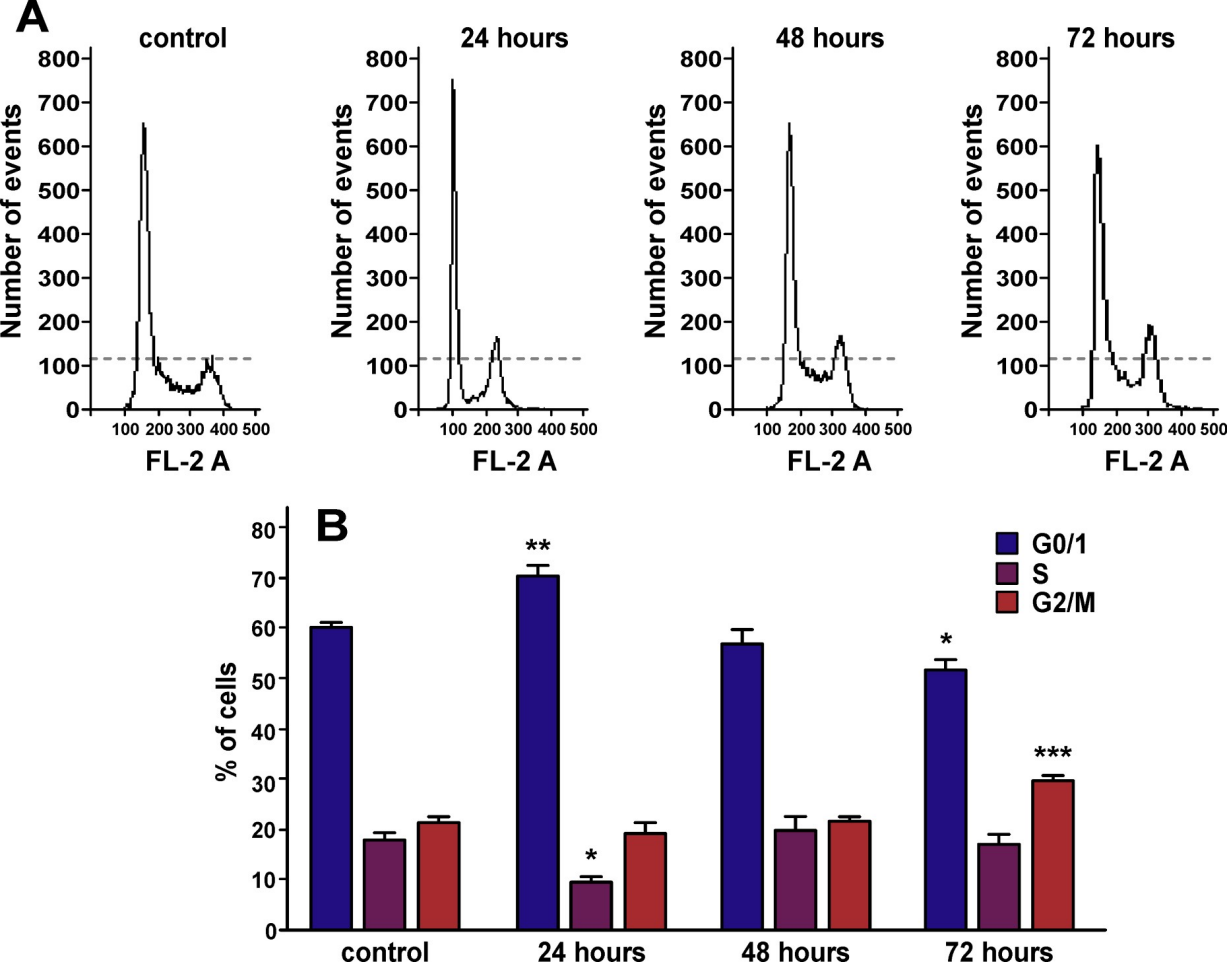
Cell cycle arrest in A549 cells treated by ws-Lynx1. (A). Representative nuclei population distributions of A549 cells before and after incubation with ws-Lynx1. (B). % of cells in the each cell cycle phase determined by Flowing Software. The data are presented as % of cells in the each cell cycle phase ± SEM, n = 6–7; * (p<0.05), ** (p<0.01), and *** (p<0.001) indicate the significant difference between the control and ws-Lynx1 treated groups by One-way ANOVA followed by a Dunnet’s *post hoc* test.

To elucidate whether ws-Lynx1 treatment induces the mitotic inhibition during cell division, we performed cell cycle analysis. FACS analysis demonstrated that the 24 h incubation of A549 cells with ws-Lynx1 significantly increased the number of cells in the G0/1 phase of cell cycle, while the number of cells in the S phase was significantly decreased (Fig 5A and 5B). Contrarily, we did not observe any difference in the cell proportion in each cell cycle phase after the 48 h incubation with ws-Lynx1, while the 72 h incubation resulted in the increased number of cells in the G2/M phase compared to the control, that accompanied by the decrease of the G0/1 cell population (Fig 5A and 5B). Thus, we can conclude that ws-Lynx1 induces the G0/1 and G2/M cell cycle arrest in A549 cells after 24 and 72 h incubation, respectively. This observation confirms the presence of the multi-step cellular response to the ws-Lynx1 treatment. The primary response to ws-Lynx1 results in the G0/1 cell cycle arrest during the 24 h incubation preventing cells from entering into the S phase, but after 48 h A549 cells could bypass this cell cycle blockade and start DNA replication. However, during the 72 h incubation, the response to secondary messengers (auto- paracrine regulators, growth factors, or cytokines) results in the G2/M cell cycle arrest, so A549 cells could not divide after successful pass of the S phase. This type of the cell cycle arrest could be directly connected with induction of apoptosis of A549 cells (see below).

### Ws-Lynx1 controls the growth of A549 cells by modulation of α7-nAChRs and activation of PKC/IP3 pathway and other signaling cascades

To study the mechanisms underlying the ws-Lynx1 action, we investigated its effect on the growth of A549 cells in the presence of inhibitors of different cell surface receptors: bicuculline (antagonist of the GABA_A_ receptors), gefitinib (inhibitor of the epidermal growth factor receptor EGFR), AG 825 (inhibitor of the ErbB2 receptor), timolol (inhibitor of the β-adrenergic receptors (β-AR)), mecamylamine (Mec, the nonspecific non-competitive antagonist of nAChRs), and methyllycaconitine (MLA, the specific α7-nAChRs antagonist). We used 1 μM ws-Lynx1 concentration, at which the maximal reduction of the cell growth was observed, and inhibitor concentrations equivalent or larger than IC_50_ values for the respective receptor. All the inhibitors (except bicuculline) alone did not significantly influence the viability of A549 cells upon 72 h incubation. Co-incubation of the cells with ws-Lynx1 and bicuculline, gefitinib, AG 825, or timolol did not diminish the ws-Lynx1 effect on the A549 cell growth (Fig 4B), while co-application with Mec or MLA canceled the ws-Lynx1 effect restoring the number of viable cells to the control level. Probably, the ws-Lynx1 action on the A549 cell growth is mediated by α7-nAChR.

**Fig 6:**
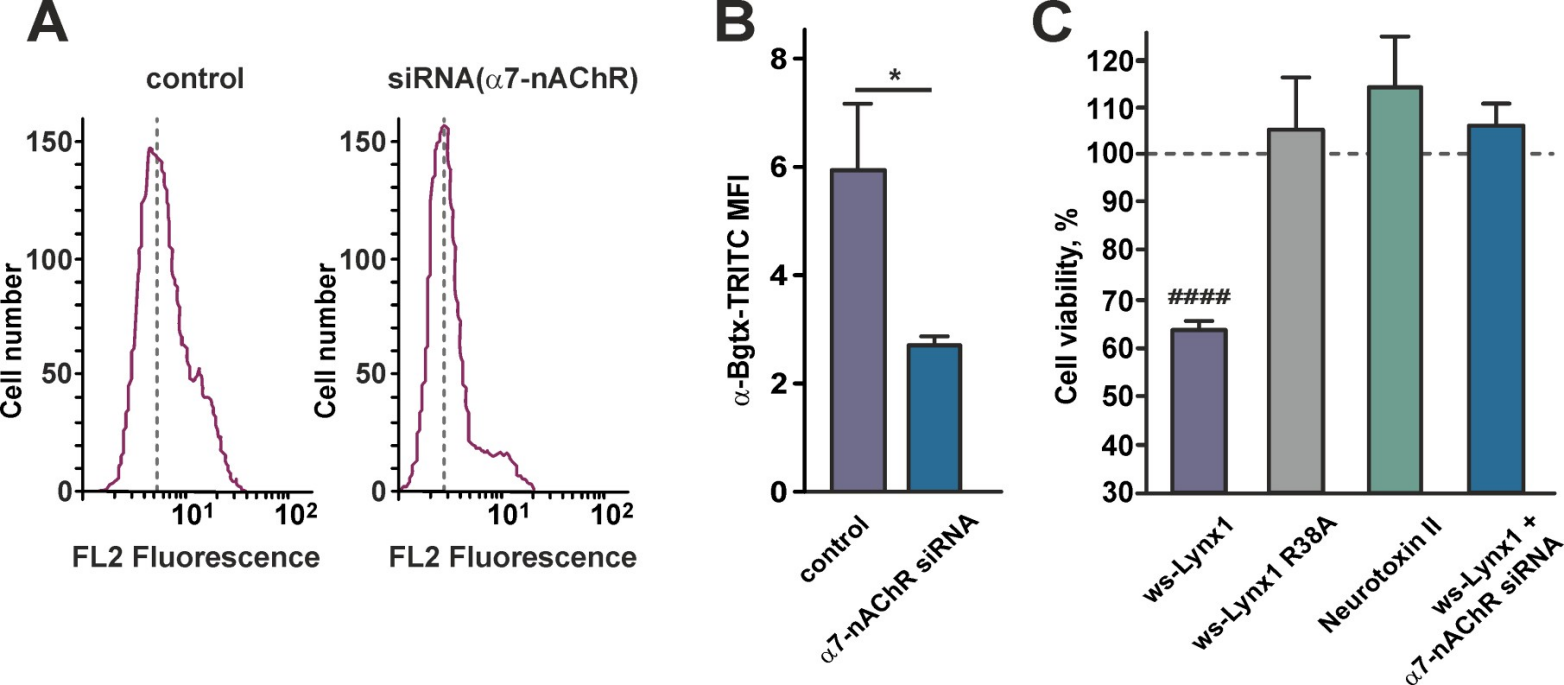
Ws-Lynx1 activity on A549 cells is mediated by interaction with α7-nAChRs. (A). Representative histograms of cell distribution according to intensity of TRITC-labeled α-bungarotoxin for untreated cells (left) and cells with the blocked α7-nAChR expression by siRNA (right). (B). Median fluorescence intensities for TRITC-labeled α-bungarotoxin in untreated cells and cells with the blocked α7-nAChR expression by siRNA. Data are presented as mean ± SEM, n = 6. * (p<0.05) indicates the significant difference between the groups by two-tailed t-test. (C). Influence of ws-Lynx1, ws-Lynx1 in the presence of siRNA against α7-nAChR, the ws-Lynx1 R38A mutant, and recombinant neurotoxin II on the viability of A549 cells. The data are mean ± SEM, n = 7–11; ^####^ (p<0.0001) indicates the significant difference from the control (100%) by a two-tailed one sample t-test. The control level (100% of viable cells) corresponds to the untreated cells and is shown by the dashed line.

To demonstrate that the antiproliferative action of ws-Lynx1 is connected with α7-nAChRs, we blocked expression of this receptor by siRNA. Flow cytometry showed that 48 h transfection of A549 cells with siRNA led to more than two-fold decrease of the functional α7-nAChRs expression on the cell surface (Fig 6A and 6B). The WST-1 assay showed that knockdown of the *α7-nAChR* gene completely cancelled the ws-Lynx1 antiproliferative effect (Fig 6C). We also tested the ws-Lynx1 R38A mutant [45] and the recombinant analogue of neurotoxin II from *Naja oxiana* [46], the proteins which have no activity against α7-nAChRs. As expected, both of these proteins did not reduce the A549 cell growth (Fig 6C). Thus, we can conclude that the ws-Lynx1 antiproliferative effect on A549 cells is a result of the protein interaction with α7-nAChRs.

The observed difference between the effects of nicotine (agonist of nAChRs), MLA (specific α7 antagonist), and ws-Lynx1 on the A549 cell viability (Fig 4B) revealed the difference in the mechanisms of action of these compounds. It seems like ws-Lynx1 acts on nAChRs in A549 cells in the unusual manner, not like an agonist or antagonist. This is indeed the case, the original study of ws-Lynx1 on α7-nAChRs expressed in *Xenopus* oocytes revealed that ws-Lynx1 by itself is unable to evoke currents through the receptor channel, but it potentiates ACh-evoked currents at ≤1 μM concentrations and suppresses them at higher concentrations [28]. Probably, ws-Lynx1 can modulate effects of agonist on the signal transduction through the α7 receptor (as shown here for nicotine), but cannot overcome the effects of antagonists, which block the receptor.

The signaling through α7-nAChRs could activate intracellular responses by two pathways. The canonical ionotropic pathway is connected with the Ca^+2^ influx through the receptor channel and subsequent activation of the Ca^2+^/calmodulin-dependent protein kinase (CaMK) [47]. The alternative metabotropic pathway is not associated with the ion flow through the receptor channel, but involves activation of the phospholipase C (PLC) and the IP_3_ receptors [48–50]. PLC hydrolyzes the membrane phospholipid phosphatidylinositol 4,5-bisphosphate with production of the two second messengers IP_3_ and 1,2-diacylglycerol (DAG). IP_3_ causes the Ca^2+^ release from the ER due to the interaction with the IP_3_ receptors [51] and both Ca^2+^ and DAG in turn activates the protein kinase C (PKC) [52]. Activation of both these pathways results in the phosphorylation of the MAP/ERK kinase that transmits the signal to the nucleus activating different transcriptional factors. Previously it was described, that ws-Lynx1 inhibits the nicotine-induced ERK phosphorylation in the striatal brain slices [53]. In addition, PKC could activate signaling through the c-Jun N-terminal kinase (JNK). The influence of ws-Lynx1 on the JNK activation in the primary cortical neurons was demonstrated recently [54].

To study the possible signaling pathways involved in the ws-Lynx1 action on the A549 cells, we used the selective inhibitors of MAP kinase MEK/ERK (PD98059), JNK (SP600125), MAP kinase p38 (SB203580), phosphoinositide 3-kinase PI3K (wortmannin), PKC (Go 6983), IP_3_ receptors (xestospongin B), and transcription factors NFκB (Bay 11–7082 and JSH-23), STAT3 (S3I-201), and STAT5 (285986-31-4). Bay 11–7082 prevents the dissociation of the inhibitory IκB kinase (IKK) from NFκB, and JSH-23 inhibits the p65 subunit nuclear translocation. PD98059, wortmannin, and Go 6983 slightly reduced the A549 cell viability upon 72 h incubation, while other inhibitors did not significantly influence the A549 cell growth. All investigated inhibitors (except S3I-201) significantly reduced or even abolished the ws-Lynx1 effect on the cancer cell growth (Fig 4C).

The observed cancelation of the ws-Lynx1 effect by the inhibitors of the IP_3_ receptors, PKC, and MEK/ERK reveals the involvement of the PLC-dependent signaling with further activation of the transcriptional factors NFκB and STAT5, but not STAT3. The dependence of the ws-Lynx1 effect on JNK, p38 MAP kinase, and PI3K indicates that other signaling pathways could also be involved. It should be noted that in contrast to other cell types, where ERK and JNK are considered as oncogenic kinases, the activation of MEK/ERK [55], JNK and p38 MAPK [56] signaling pathways is required for triggering of cytostatic or apoptotic effects in A549 cells.

### Ws-Lynx1 regulates phosphorylation of kinases and transcription factors which control the cell growth

**Fig 7:**
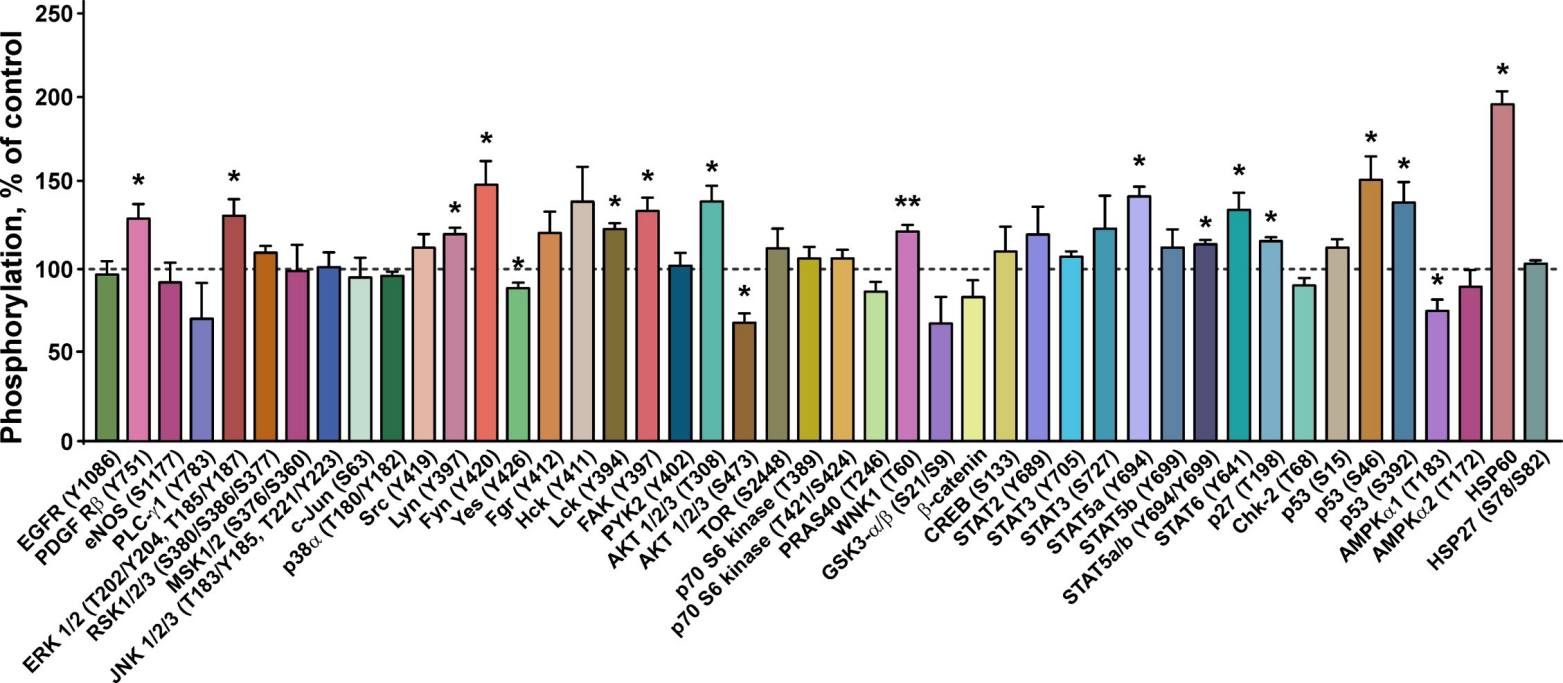
Influence of ws-Lynx1 on phosphorylation of intracellular kinases and regulatory proteins in A549 cells. The phosphorylation level is presented as % of the control (100%, untreated cells, dashed line), n = 4; * (p<0.05) and ** (p<0.001) indicate the significant difference from the control by a two-tailed one sample t-test.

To further disclose signaling cascades triggered by ws-Lynx1 in A549 cells, we studied the phosphorylation of the set of 45 kinases and regulatory proteins upon the 72 h cell incubation with 1 μM of ws-Lynx1 (Fig 7). In agreement with the results of inhibitory analysis (Fig 4C), we observed the significant increase in the ERK1/2 phosphorylation at the positions T202, Y204, T185, and Y187 upon the ws-Lynx1 treatment (Fig 7). Contrary to ERK, we did not detect an increase in the phosphorylation level of JNK1/2/3 (T183, Y185, T221, Y223) and p38α (T180, Y182) (Fig 7), although the inhibitory analysis revealed the participation of these kinases in the ws-Lynx1-induced cell growth suppression (Fig 4C). Probably, the phosphorylation level of JNK and p38α does not change upon the ws-Lynx1 treatment and is already sufficient to activate downstream players of their cascades. In line with this hypothesis, the constant JNK and p38 activity was previously shown in A549 cells [57,58].

**Fig 8:**
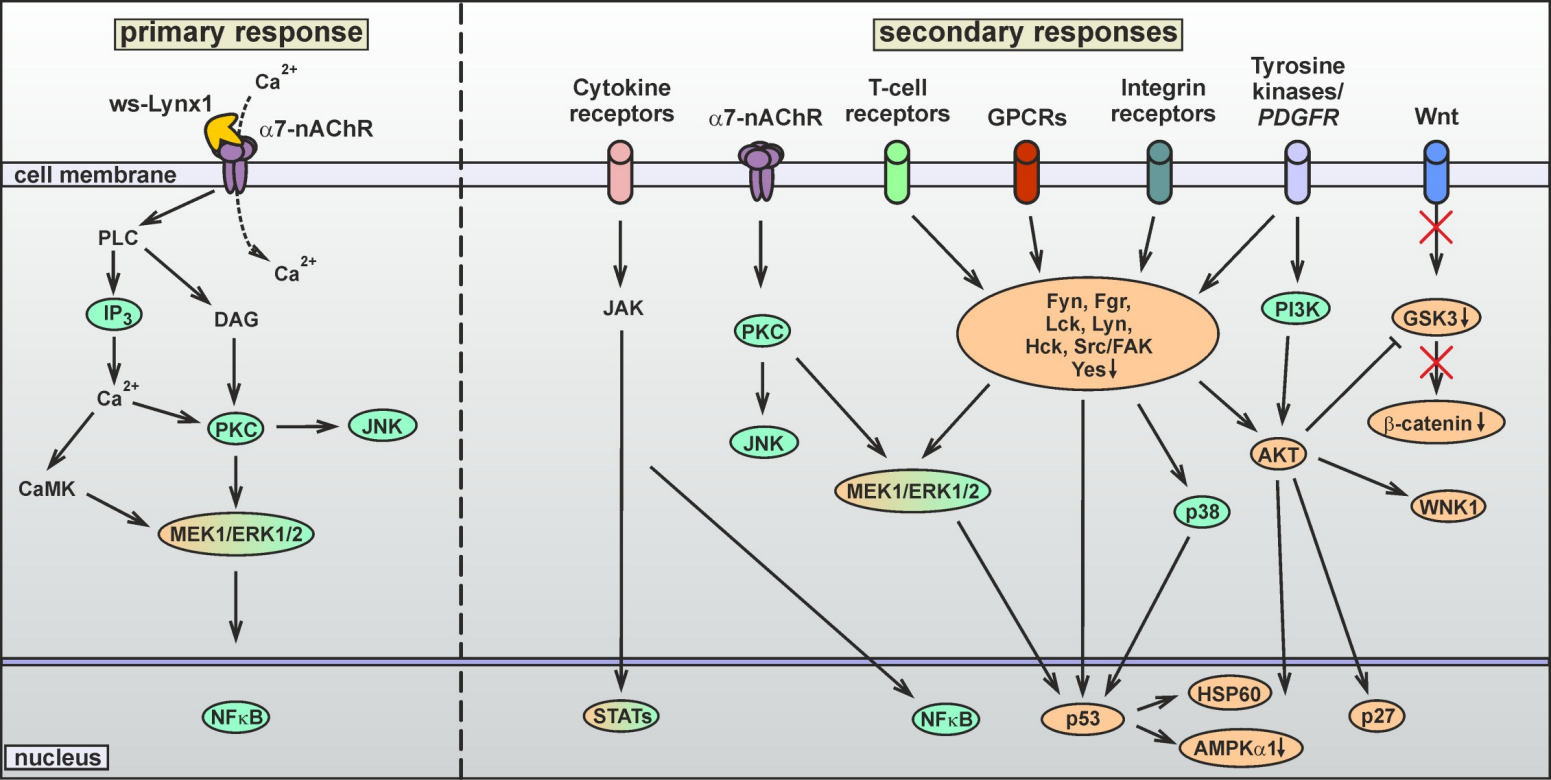
Proposed two-stage mechanism of the ws-Lynx1 action in A549 cells. On the first stage, ws-Lynx1 interacts with α7-nAChRs on the cell membrane. This activates the PLC (PKC/IP3) and ERK signaling pathways, the transcription factor NFkB, and induces the gene transcription of auto- paracrine regulators (growth factors, cytokines etc). The secondary effects of ws-Lynx1 are mediated by different membrane receptors activated by these regulators. On the second stage, the JAK, JNK, p38α, Src, PI3K, and AKT kinases are activated. This results in the activation of the transcription factors, the proapototic factor p53, and mislocalization of the p27 protein. Proteins colored in green were revealed by an inhibitory analysis, and ones colored in orange–by a phosphorylation assay.

As described above, the cell cycle analysis demonstrated that ws-Lynx1 causes two types of the cell cycle arrest–initial G0/1 arrest and G2/M arrest after 24 and 72 h incubations, respectively. Thus, the prolonged ws-Lynx1 application could result in the secondary response(s) related with the activation of different membrane receptors, including growth factors receptors, integrins, G-protein-coupled receptors (GPCRs), T-cell receptors etc. The intracellular signaling cascades triggered by these receptors are mediated by different kinases from the Src family (Fig 8). Indeed, we observed the increased phosphorylation of the platelet-derived growth factor receptor β (PDGFRβ) at the position Y751 and changes in the phosphorylation level of different kinases from the Src family (Fig 7). The significant increase in the phosphorylation of Lyn(Y397), Fyn(Y420), FAK(Y397), and Lck(Y394) and decrease in the Yes(Y426) phosphorylation were observed upon the ws-Lynx1 treatment (Fig 7). In addition, the enhanced phosphorylation of the Src(Y419), Fgr(Y412), and Hck(Y411) kinases was also detected, but did not reach statistical significance. Some of the Src kinases are involved in control of cell growth, proliferation, and adhesion, and are considered as oncogenic. For example, the Fyn and Fgr kinases were significantly down-regulated in patients with lung carcinoma compared to normal cells [59]. The Fgr silencing enhances the phosphorylation of the protein kinase B (AKT) at the Ser473 position, resulting in the AKT stabilization in ovarian carcinoma [60]. The Yes kinase promotes the growth of basal-like breast cancers [61]. Thus, the reverse phosphorylation pattern observed under the ws-Lynx1 treatment (Fig 7) could be responsible for the suppression of cancer cell growth. The FAK and Fyn kinases participate in the regulation of adhesion of colon cancer cells [62].

**Fig 9:**
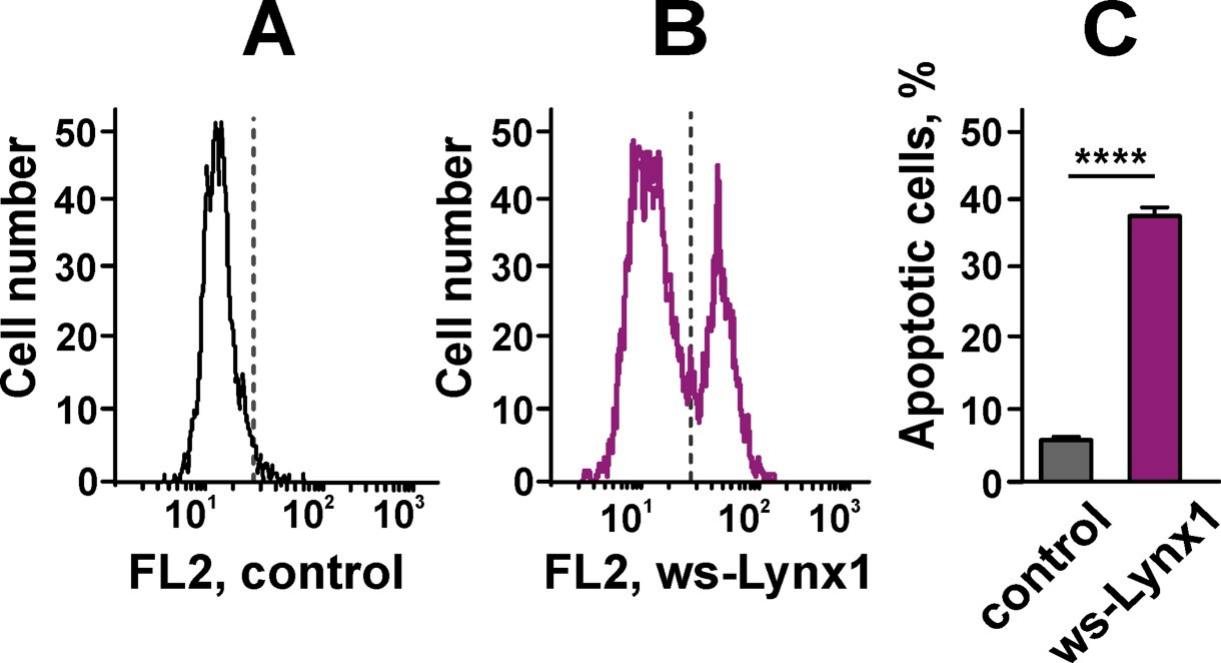
Externalization of phosphatidylserine in A549 cells after 72 h incubation with ws-Lynx1. (A). The representative histogram of flow cytometry after Annexin V-PE staining of the control cells. Grey line shows threshold for the Annexin V positive cellular phenotype. X axis shows PE fluorescence (FL2 channel). (B). The representative histogram of flow cytometry after Annexin V-PE staining of the ws-Lynx1-treated cells. Grey line shows threshold for the Annexin V positive cellular phenotype. X axis shows PE fluorescence (FL2 channel). (C). Bars represent the percent of cells with the externalized phosphatidylserine in the control and ws-Lynx1-treated cells. The data are presented as % of cells in the gate, n = 8, **** (p<0.0001) indicates the significant difference of the groups from each other by a two-tailed t-test.

The growth factor receptors could also be responsible for the observed changes in the AKT phosphorylation (Figs 7 and 8). This signaling cascade could be mediated by PI3K, whose inhibition abolished the ws-Lynx1 effect (Fig 4C). Contrary, the involvement of the ERK signaling in the AKT activation is unlikely, as we did not observe changes in the phosphorylation of mitogen and the stress activated protein kinase MSK1/2 (S376, S360), which transfers the signal between ERK and AKT (Fig 7). The application of ws-Lynx1 increased the AKT phosphorylation at the T308 position and decreased the phosphorylation of Ser473 (Fig 7). The AKT phosphorylation in these positions causes activation and stabilization of active AKT, respectively [63]. So we cannot say exactly, if the ws-Lynx1 treatment enhanced or not the AKT activity in A549 cells. At the same time, we observed the significant increase in the phosphorylation of the cyclin-dependent kinase inhibitor p27(T198), which could be connected with the AKT activation. This regulator of the cell cycle is considered to be a tumor suppressor and is often inactivated via impaired synthesis, accelerated degradation, or by mislocalization. The p27 phosphorylation induces the p27 mislocalization from the nucleus to the cytoplasm where it undergoes the proteasomal degradation [64]. In line with this, the silencing of p27 was shown to reduce the cell proliferation and induce apoptosis in A549 cells [65]. So the ws-Lynx1-induced p27 mislocalization and further degradation in the cytoplasm may also be involved in control of the A549 cell growth. The AKT activation could also suppress the glycogen synthase kinases-3α/β (GSK3-α/β),–a participant of the Wnt-signaling cascade. Indeed, we observed the moderate (non significant) decrease of the phosphorylation of GSK3-α/β (S9, S21) and β-catenin,–a protein involved in the Wnt-signaling downstream to GSK3-α/β (Fig 9). So, we can speculate, that the ws-Lynx1 effects are not connected with the Wnt-signaling. Another kinase, which phosphorylation level was increased and which can be activated by AKT, is the WNK1 kinase (Fig 7).

The activation of the transcriptional factor NFκB, which according to the inhibitory analysis participates in the ws-Lynx1 effect on the A549 cell growth (Fig 4C), may be mediated by the activation of ERK1/2 and/or AKT (Fig 8). In line with the results of the inhibitory analysis, we observed the significant enhancement of the phosphorylation of the transcription factors STAT5a(Y694) and STAT6(Y641), but not of STAT3(Y705, S727) (Fig 7). Although STAT5 could be activated by the Src kinases, the simultaneous activation of the several STATs is probably connected with the activation of the cytokine receptors and downstream JAK kinases (Fig 8). This signaling pathway could be responsible for some of the effects induced by ws-Lynx1. For example, the dephosphorylated STAT6 drives the constitutive cyclooxygenase-2 (COX2) expression and cytotoxicity resistance in lung cancer cells [66], so the ws-Lynx1-induced STAT6 phosphorylation could diminish the COX2 level and increase the sensitivity of A549 cells to apoptosis. Despite the quite speculative nature of the proposed ws-Lynx1 action model, we can conclude that ws-Lynx1 controls the growth of A549 cells by activation of different intracellular signaling pathways either immediately after initial interaction with α7-nAChR or by inducing secondary responses associated with secretion of different signaling factors.

### Ws-Lynx1 induces apoptosis in A549 cells via phosphorylation of the proapoptotic factor p53

The large increase in the phosphorylation of the tumor protein p53, essential for the apoptosis induction [67], was observed upon the A549 cell treatment with ws-Lynx1 (Fig 7). The phosphorylation of p53 at the Ser46 and Ser392 positions is characteristic for the active p38 and ERK1/2 kinases, respectively [68,69]. The p53 phosphorylation could also be controlled by the Lck and Lyn kinases from the Src family [70,71]. The triggering of apoptotic signaling is supported by the large increase in the phosphorylation of the heat shock protein 60 (HSP60, Fig 7). HSP60 initiates events upstream of the cytochrome C-triggered apoptosome activation and participates in the caspase-3 activation [72]. In addition, we observed the decrease in the phosphorylation of the other p53 signaling partner–the AMPKα1 kinase at the position T183, which is responsible for the control of cell proliferation (Fig 7). Thus, the p53 activation by ERK, p38, and/or Src kinases could trigger apoptosis in A549 cells in response to the ws-Lynx1 application.

To prove this, we used the Annexin V-PE conjugate that binds to the externalized phosphatidylserine, an early apoptotic marker. The analysis by flow cytometry revealed that the number of A549 cells with the externalized phosphatidylserine significantly increased upon the 72 h treatment with 1 μM ws-Lynx1 from 5.39 ± 0.6% to 37.44 ± 2.16% as compared to the control (Fig 9). Thus, ws-Lynx1 down-regulates the growth of A549 cells not only by suppression of proliferation via the cell cycle arrest, but also by the apoptosis induction. Notably, we did not observe DNA fragmentation in nuclei of A549 cells stained by Hoechst 33342 after the 72-hour incubation with ws-Lynx1. It should be mentioned, that the assay based on the morphology analysis of nuclei stained by Hoechst dye detects the changes characteristic for the late stages of apoptosis, while the phosphatidylserine externalization is a marker of early apoptosis [73]. In line with it, no significant increase in the p53 phosphorylation at the Ser15 position associated with the DNA damage on the late stages of apoptosis was observed (Fig 7).

## Conclusion

nAChRs play an important role in many essential processes and are involved in nicotine-induced carcinogenesis [6]. Previously we demonstrated that the human secreted proteins SLURPs could inhibit the growth of cancer cells of epithelial origin [18,19]. Human Lynx1 was also found to participate in the regulation of the lung cancer cells growth [26,44]. Here, for the first time we described the Lynx1 protein expression in epidermal, colon and breast epithelial cells. Colocalization of Lynx1 with α7-nAChRs in lung and colon cancer cells was observed. We also found that the water-soluble variant of human Lynx1 controls the growth and apoptosis of non-small lung cancer cells. Based on the data obtained, we propose the two-stage mechanism of the ws-Lynx1 action (Fig 7). Ws-Lynx1 interaction with α7-nAChRs activates different intracellular kinase cascades with the subsequent phosphorylation of the proapototic factor p53 and other factors participated in the control of the gene transcription as well as cell growth and apoptosis. Thus, the endogenous Ly6/uPAR proteins like Lynx1 are the promising prototypes for development of new drugs for treatment of epithelial cancers.

## Supporting information

S1 Fig Analysis of unspecific binding of the secondary antibodies in cortical neurons, oral Het-1A keratinocytes and HEK-293T cells. Cells were sequentially incubated with EBSS and with secondary anti-rabbit Alexa-488 labeled IgG (green) and anti-mouse Alexa-594 labelled antibodies (red). Cell nuclei were visualized by Hoechst 33342 (blue). Scale bar 10 μm.
(TIF)

S2 Fig Analysis of unspecific binding of the secondary antibodies in A549, HT-29, MCF-7, and A431 cells. Cells were sequentially incubated with EBSS and with the secondary anti-rabbit Alexa-488 labeled IgG (green) and anti-mouse Alexa-594 labelled antibodies (red). Cell nuclei were visualized by Hoechst 33342 (blue). Scale bar 10 μm.
(TIF)

S3 Fig Analysis of anti-Lynx1 antibodies specificity. Anti-Lynx1 antibodies was incubated with ws-Lynx1 for 1 h, and A549 cells were stained either with rabbit anti-Lynx1 and mouse anti-α7-nAChR antibodies or by the mixture of ws-Lynx1 with rabbit anti-Lynx1 and mouse anti-α7-nAChR antibodies. Secondary antibodies were anti-rabbit Alexa-488 labeled IgG (green) and anti-mouse Alexa-594 labelled antibodies (red). Cell nuclei were visualized by Hoechst 33342 (blue). Scale bar 10 μm.
(TIF)

S1 Table Primers used for real-time PCR. (DOCX)
