# Supplementary figures and images for: Water-soluble variant of human Lynx1 induces cell cycle arrest and apoptosis in lung cancer cells via modulation of α7 nicotinic acetylcholine receptors
Source: PLoS One. 2019 May 31;14(5):e0217339. doi: 10.1371/journal.pone.0217339 (PMC6544245; doi:10.1371/journal.pone.0217339)

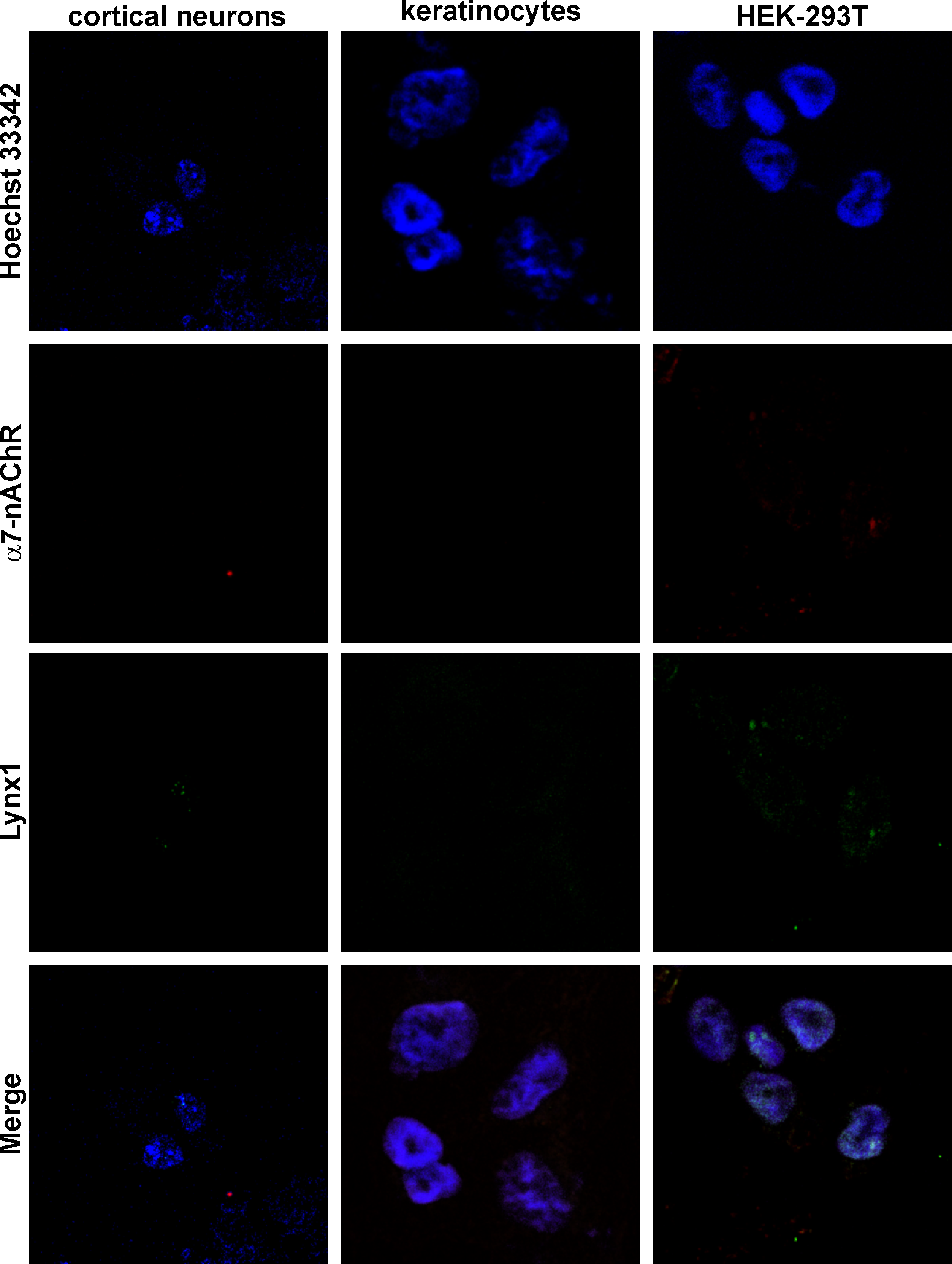

Supplement: S1 Fig — Cells were sequentially incubated with EBSS and with secondary anti-rabbit Alexa-488 labeled IgG (green) and anti-mouse Alexa-594 labelled antibodies (red). Cell nuclei were visualized by Hoechst 33342 (blue). Scale bar 10 μm. (TIF) [file pone.0217339.s001.tif]

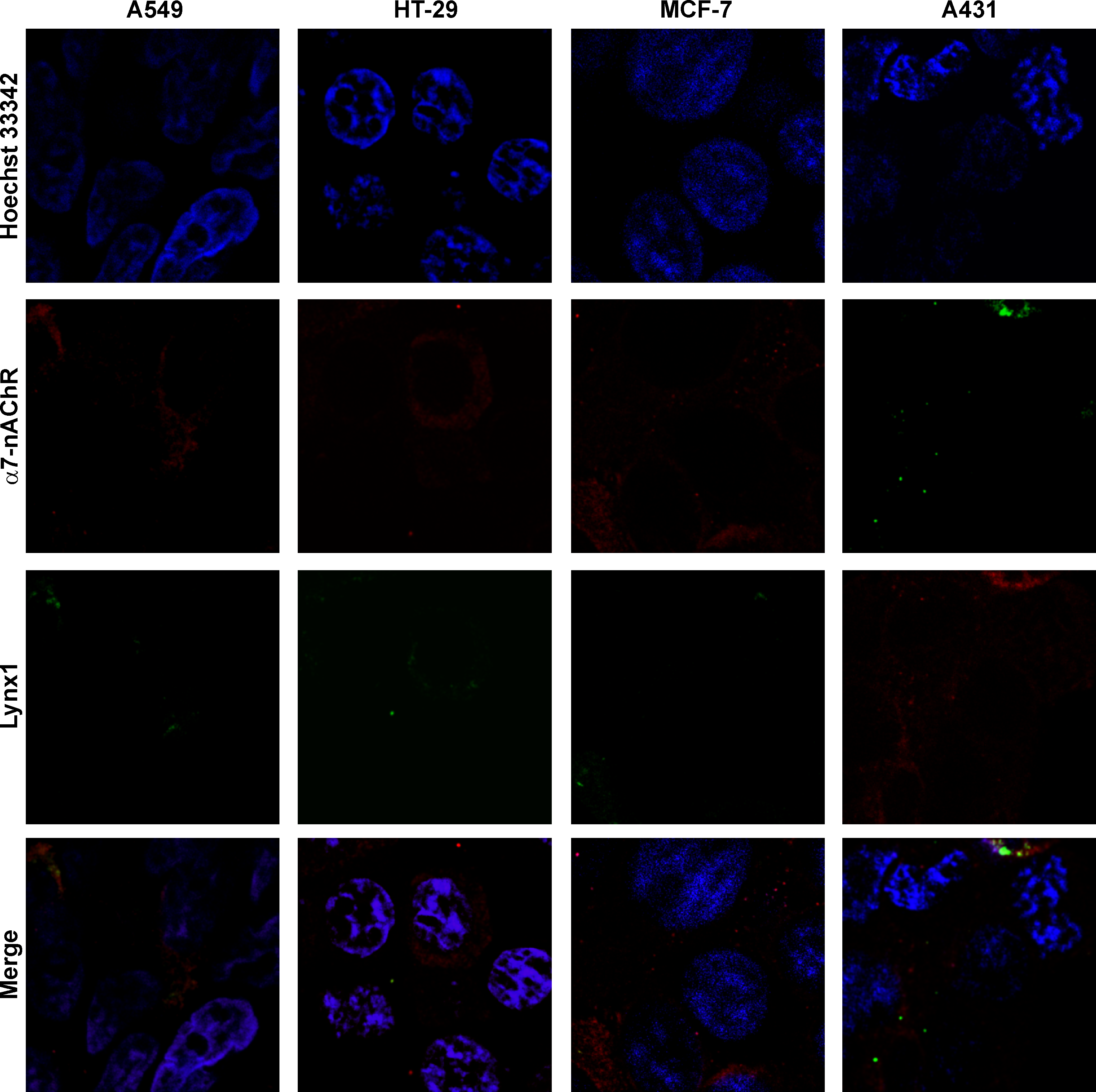

Supplement: S2 Fig — Cells were sequentially incubated with EBSS and with the secondary anti-rabbit Alexa-488 labeled IgG (green) and anti-mouse Alexa-594 labelled antibodies (red). Cell nuclei were visualized by Hoechst 33342 (blue). Scale bar 10 μm. (TIF) [file pone.0217339.s002.tif]

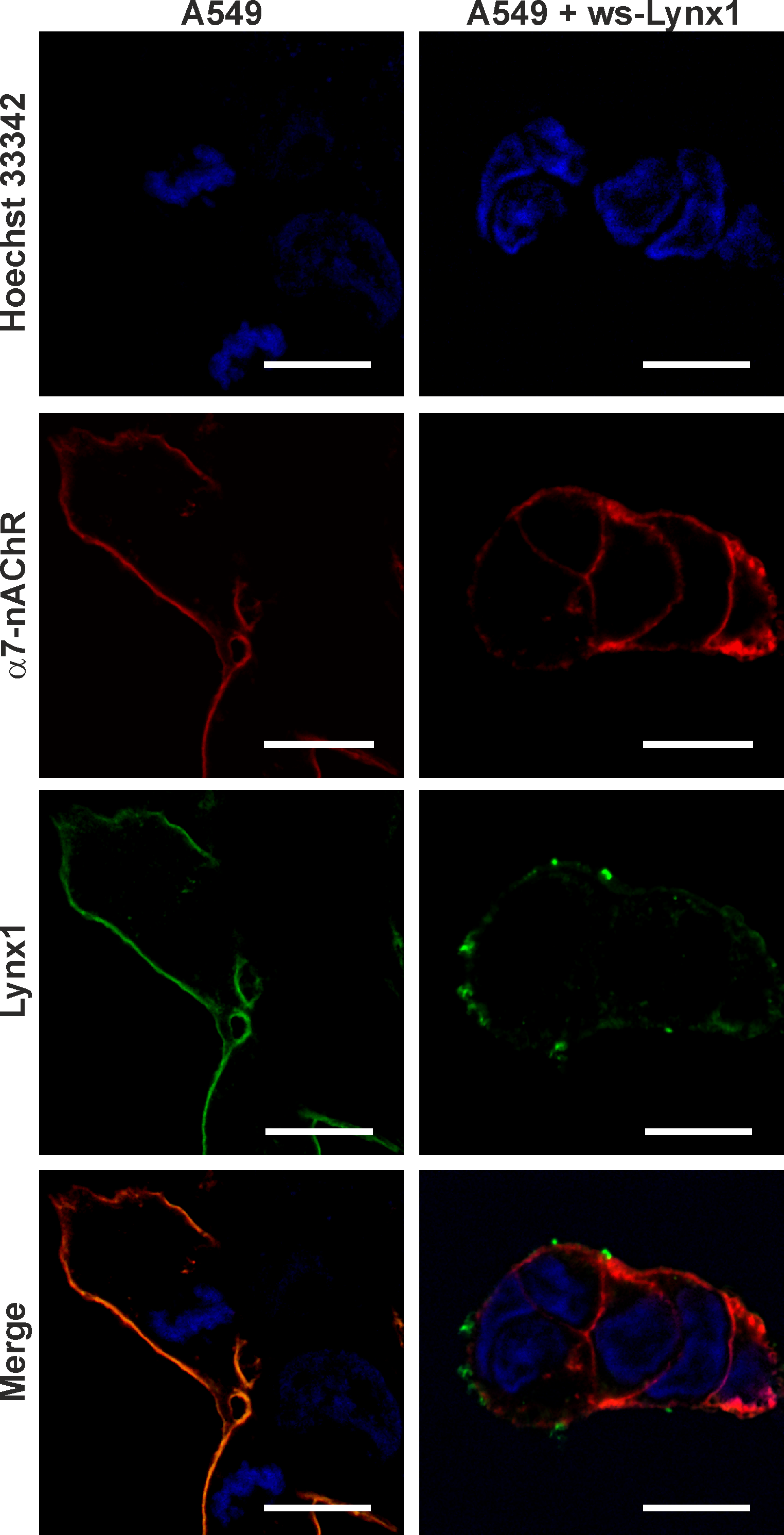

Supplement: S3 Fig — Anti-Lynx1 antibodies was incubated with ws-Lynx1 for 1 h, and A549 cells were stained either with rabbit anti-Lynx1 and mouse anti-α7-nAChR antibodies or by the mixture of ws-Lynx1 with rabbit anti-Lynx1 and mouse anti-α7-nAChR antibodies. Secondary antibodies were anti-rabbit Alexa-488 labeled IgG (green) and anti-mouse Alexa-594 labelled antibodies (red). Cell nuclei were visualized by Hoechst 33342 (blue). Scale bar 10 μm. (TIF) [file pone.0217339.s003.tif]
