# Supplementary material for: Water-soluble variant of human Lynx1 induces cell cycle arrest and apoptosis in lung cancer cells via modulation of α7 nicotinic acetylcholine receptors
Source: PLoS One. 2019 May 31;14(5):e0217339. doi: 10.1371/journal.pone.0217339 (PMC6544245; doi:10.1371/journal.pone.0217339)
Supplement: S1 Table — (DOCX) [file pone.0217339.s004.docx]

**S1 Table**. Primers used for real-time PCR.

| gene | primer sequence | | Amplicon, bp |
| --- | --- | --- | --- |
|  | forward | reverse |  |
| ***β-actin*** | TCATGTTTGAGACCTTCAACAC | GTCTTTGCGGATGTCCACG | 250 |
| ***Lynx-1*** | ACCACTCGAACTTACTTCACC | ATCGTACACGGTCTCAAAGC | 81 |
| ***α3-nAChR*** | CCTGTTCCAGTACCTGTTCG | AGACATGGACACCTCAAACTG | 97 |
| ***α4-nAChR*** | GTCAAAGACAACTGCCGGAGACTT | TGATGAGCATTGGAGCCCCACTGC | 300 |
| ***α7-nAChR*** | TGCACGTGTCCCTGCAAGGC | GTACACGGTGAGCGGCTGCG | 112 |
| ***α9-nAChR*** | ATCCTGAAGTACATGTCCAGGATC | TGGCCTTGTGGTCCTTGAGGCACT | 300 |
| ***β2-nAChR*** | ACGGTGTTCCTGCTGCTCATC | CACACTCTGGTCATCATCCTC | 507 |
